# Dosimetric implications of the potential radionuclidic impurities in ^153^Sm-DOTMP

**DOI:** 10.1016/j.apradiso.2022.110246

**Published:** 2022-04-14

**Authors:** Richard E. Wendt, Alan R. Ketring, R. Keith Frank, Jaime Simón

**Affiliations:** a Department of Imaging Physics, The University of Texas MD Anderson Cancer Center, Unit 1352, 1515 Holcombe Blvd, Houston, TX, 77030, USA; b IsoTherapeutics Group, LLC, 1004 S. Velasco St, Angleton, TX, 77515, USA

**Keywords:** Internal dosimetry, Radionuclidic impurities, ^153^Sm-DOTMP

## Abstract

Thehuman internal dosimetry of the radionuclidic impurities of samarium-153 in a new bone-seeking radiopharmaceutical, ^153^Sm-1,4,7,10tetraazacyclododecanetetramethylenephosponic acid (^153^Sm-DOTMP), has been estimated from preclinical data. The effective dose from the impurities in lower-specific-activity ^153^Sm is less than 17% of the effective dose from pure Sm-153. It has a background-equivalent radiation time for a dosage of 37 MBq/kg of less than one-half year.

## 1. Introduction

A new radiopharmaceutical, ^153^Sm-labeled 1,4,7,10tetraazacyclododecanetetramethylenephosponic acid (PubChem, 2022) (^153^Sm-DOTMP), is currently undergoing clinical trials for the treatment of bone cancer and bone cancer metastases. It is prepared using lower-specific-activity ^153^Sm. An estimate of its human internal dosimetry that was based upon preclinical data and the properties of ^153^Sm demonstrates that its biodistribution, which is primarily long-term uptake in the skeleton, imparts a radiation absorbed dose predominantly to the bone, the red marrow, and the urinary bladder (Simon et al., 2012). Samarium-153 is known to have long-lived radionuclidic impurities (Ma et al., 1996; Kalef-Ezra et al., 2015; Naseri et al., 2021). These arise from its production by neutron activation of enriched stable ^152^Sm, which may still contain small amounts of several other isotopes and elements, as well as subsequent activation of its daughter, ^153^Eu, and of the activation products themselves (Ma et al., 1996; Kalef-Ezra et al., 2015). The DOTMP chelant binds these impurities with the same high degree of efficiently as it does ^153^Sm (Simon et al., 1991). Thus, their contributions to the internal dosimetry of ^153^Sm-DOTMP are both an important consideration clinically, given their persistence in the skeleton, and a relatively straightforward question to answer, given estimates of the uptake and clearance of ^153^Sm-DOTMP in various source organs.

## 2. Materials and methods

The significant radionuclidic impurities of reactor-produced ^153^Sm are tabulated in Table 1 (Kalef-Ezra et al., 2015; Naseri et al., 2021). The relative concentrations of the radionuclidic impurities in four sources of ^153^Sm – activation of naturally abundant and of enriched ^152^Sm as reported by Naseri et al. (Naseri et al., 2021), and activation of higher-specific-activity (40.8 GBq/mg) and of lower-specific-activity (8.77 GBq/mg) ^153^Sm using 99% or more enriched ^152^Sm by the University of Missouri Research Reactor (MURR) in Columbia, Missouri (Ma et al., 1996) as measured by the authors – are given in Table 2. The neutron flux and activation time for the Naseri samples are inferred from that same institution’s earlier report of producing ^153^Sm with a specific activity of 12.8 GBq/mg (Naseri et al., 2011).

A dose of ^153^Sm-ethylenediamine tetra(methylene phosphonic acid) (^153^Sm-EDTMP, tradename Quadramet) that had been made with higher-specific-activity ^153^Sm from MURR and had been calibrated for 5.55 GBq (150 mCi) on January 7, 2009 with a stated expiration 56 h after calibration was analyzed 157 months after calibration. A sample of lower-specific-activity ^153^Sm was obtained by the authors from MURR and allowed to age for 64 days (or 33 half-lives of ^153^Sm) by which time the initial activity of the ^153^Sm had decayed by ten orders of magnitude so that it would not interfere with the peaks of the long-lived impurities.

The impurities in the MURR samples were measured using high resolution gamma spectroscopy. This was performed with a germanium crystal detector [Canberra GC2519, Mirion Technologies, Meriden, CT] coupled to a multichannel analyzer [Easy-MCA, Ortec Advanced Measurement Technology, Oak Ridge, TN]. Gammavision software [Version 7.02.01, Ortec Advanced Measurement Technology, Oak Ridge, TN] was used to analyze the spectra. The system was calibrated for energy and efficiency with a NIST-traceable multi-nuclide source. The samples were then counted for 4800 s at a distance of 11.4 cm from the detector. The activities of ^153^Gd and ^156^Eu in the higher-specific-activity sample had to be estimated based upon the measured activities of the other isotopes of europium and the activation parameters for they had decayed to an undetectable level because of their relatively short half-lives compared to the age of the sample.

The standard Medical Internal Radiation Dose (MIRD) schema (Loevinger et al., 1991) was employed in this study. A preclinical investigation of ^153^Sm-DOTMP in rats has previously been reported by the authors (Simon et al., 2012). That study was conducted with the approval of the IsoTherapeutics Group LLC Animal Care and Use Committee. The raw data from that study are decay-corrected, biological clearance data. Those data have been re-analyzed in the present study by applying the physical decay of each radionuclide and then fitting curves to the decayed data for each radionuclide. The parameters of these fits are given in the appendix. The time-integrated activity coefficients, or residence times, which represent the areas under the normalized time-activity curves, were converted to human values through Equation (8) in (Macey et al., 2001) using the murine organ masses from the earlier report and the organ masses of the ICRP 89 (International Commission on Radiological Protection, 2002) Adult Male and Adult Female models, which are the most up-to-date models that are incorporated into OLINDA/EXM Version 2.2 (Stabin et al., 2005; Stabin and Farmer, 2012). These time-integrated activity coefficients are given for each source organ and radionuclide in Table 3 for the Adult Male model and in Table 4 for the Adult Female model.

The present report takes a few departures from the original analysis of the raw data (Simon et al., 2012) in the determination of the time-integrated activity coefficients.

The time-activity curves were integrated for fifty years rather than to infinity in order to be consistent with the definition of committed dose equivalent by the US Nuclear Regulatory Commission (US Nuclear Regulatory Commission, 2021).

When the fits to the biological data that had been decayed with the physical half-life of a particular radionuclide yielded an effective half-life that exceeded the physical half-life of that radionuclide, its physical half-life was used as the effective half-life.

**Fig. 1.**
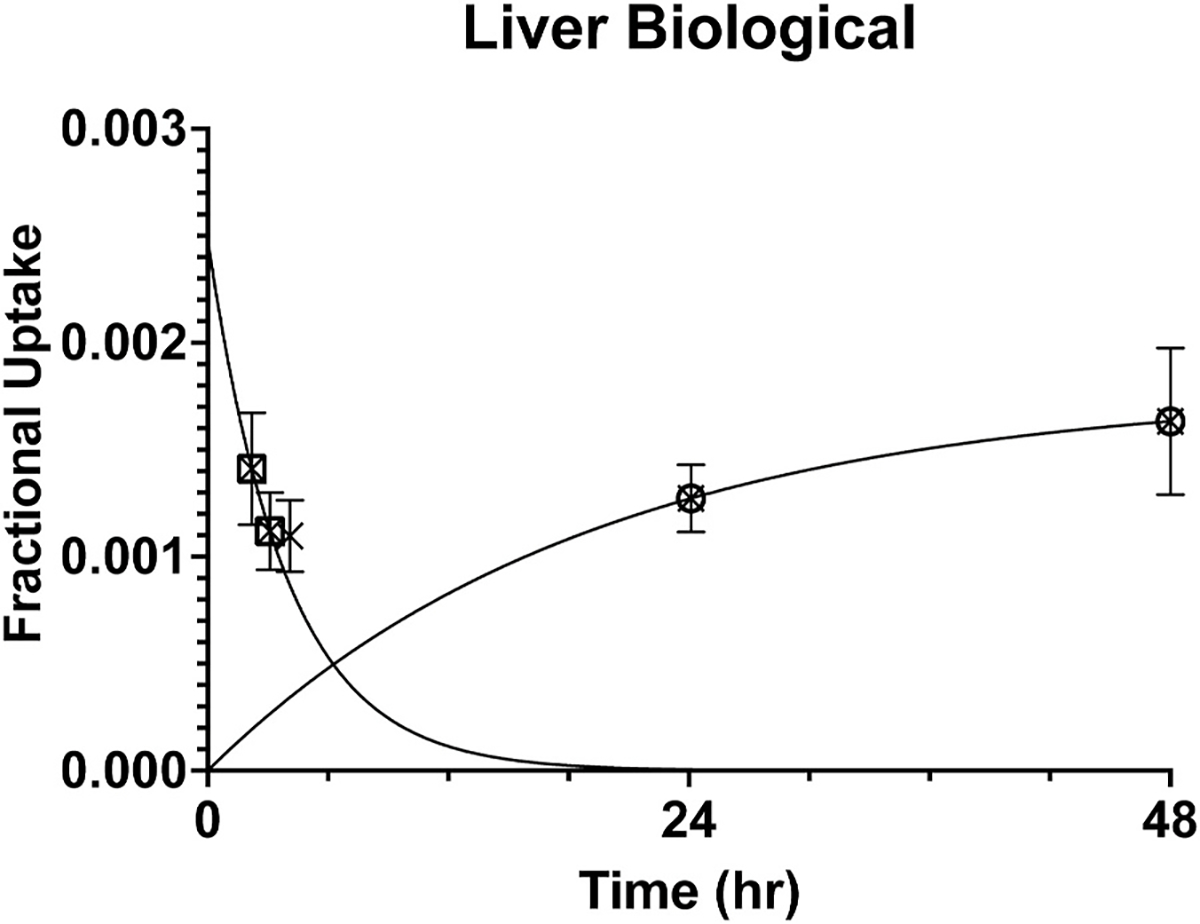
The decay-corrected uptake of ^153^Sm-DOTMP in the rat liver as a fraction of the administered activity (Simon et al., 2012). Those data are shown as crosses. A single exponential decay curve was constrained to approach zero asymptotically and was fit to the first two data, shown as squares. An uptake curve was constrained to have a nil value at time zero and was fit to the last two data shown as circles. These fitted curves illustrate the postulated rapid clearance of the blood pool that perfused the liver followed by the later accumulation in the liver of a small amount of free ^153^Sm.

The biological data for the liver have a unique time course among the source organs. They suggest a rapid clearance combined with a slow uptake as shown in Fig. 1. This is consistent with the rapid clearance of ^153^Sm-DOTMP from the blood along with the accumulation of unchelated ^153^Sm by the liver (O’Mara et al., 1969; Goeckeler et al., 1987; Banerjee et al., 2005). Despite the assumption of the longest possible biological half-life from that last datum onward, the uptake in the liver is a miniscule fraction of the total administered activity. The asymptotic value of the superimposed uptake curve in Fig. 1 is 0.18% of the administered activity, which illustrates the high binding efficiency of the DOTMP chelator even at the very low chelant-to-metal ratio of 1.5:1 that was used in this preclinical study (Simon et al., 2012). The biological time-activity data from the liver were analyzed by decaying them for each radionuclide, calculating the area under the curve out to the last datum (that is, the 48-h measurement) by a trapezoidal fit, and assuming single exponential decay with the physical half-life of the radionuclide from that time onward.

The intestines and the stomach had no appreciable activity remaining in the 48-h datum, hence just the area under the trapezoidal fit was used for those two source organs.

The rat-to-human organ mass-based conversion of the time-integrated activity coefficients was done separately for the ICRP 89 Adult Male and Adult Female models. The time-integrated activity coefficients for blood and muscle were combined into the remainder of the body term. It is not practical in rat studies to get true whole-body count data from which a whole-body time-integrated activity coefficient could be derived for the calculation of the time-integrated activity coefficient of the remainder of the body. The time-integrated activity coefficient of the large intestine was apportioned one-quarter each to the right colon and rectum and one-half to the left colon. The time-integrated activity coefficient of the skeleton was apportioned 38% to the cortical bone and 62% to the trabecular bone (Breitz et al., 2006).

## 3. Results

The time-integrated activity coefficients were analyzed with the OLINDA/EXM software [Version 2.2, Hermes Medical Solutions, Stockholm, Sweden] The resulting equivalent doses to the target organ for the ICRP 89 Adult Male and Adult Female models are given in Table 5 and Table 6. The equivalent doses to each target organ from ^153^Sm including the impurities in each of the preparations and the equivalent dose to each target organ from just the impurities alone in each preparation are given in Table 7 and Table 8.

## 4. Discussion

Although the doses per unit activity from the impurities are often higher than those from ^153^Sm, as shown in Tables 5 and 6, the small activities of the impurities compared to that of ^153^Sm make their actual effects much weaker in practice, where they are often one or two orders of magnitude less, as shown in Tables 7 and 8.

Three target organs are especially affected by the charged particle emissions of ^153^Sm: the osteogenic cells, the red marrow, and the urinary bladder wall. This situation arises from the accumulation of about 40% of the administered activity in the skeleton with an effective half-life that is close to the physical half-life while the remainder of the administered activity is cleared rapidly through the urinary system. The doses from the impurities are highest in the two target organs that receive the highest doses from ^153^Sm, namely the osteogenic cells and the red marrow. This is because of the long biological half-life of the uptake in the skeleton, which was treated as infinite.

The relatively short physical half-life of ^153^Sm compared to those of the impurities means that the activity of the impurities will grow compared to that of ^153^Sm as the preparation ages. The US Pharmacopeia limits the activity of ^154^Eu to 93 ppm of that of ^153^Sm (i. e., 0.093 μCi of ^154^Eu per mCi of ^153^Sm) in ^153^Sm-EDTMP. It limits the sum of the activities of all other radionuclidic impurities to 0.1907% of that of the ^153^Sm (US Pharmacopeia, 2013) The rationale for these values is unknown to the authors, but at these limits, the effective dose from ^154^Eu would be 16.0% of that from pure ^153^Sm in the Adult Male model and 16.6% of that from pure ^153^Sm in the Adult Female model. The times to reach these limits for the four preparations of ^153^Sm are given in Table 9. It is noteworthy that the stated expiration of the dose of ^153^Sm-EDTMP that had been prepared with higher-specific-activity ^153^Sm was 56 h after calibration, whereas the analysis of that sample suggests that it should have been only 28 h after calibration. The authors of this report postulate that the expiration time of commercially-prepared ^153^Sm-EDTMP is a standard one that was derived from the analysis of some samples of higher-specific-activity ^153^Sm that had been performed during the original development of the drug. The actual activities of the impurities are impossible to measure by any practical means during the preparation of a particular dose of a^153^Sm-labeled radiopharmaceutical.

The authors have found only one previous report of the measurement of ^153^Gd in a sample of ^153^Sm (Nuñez et al., 2015) although its presence is not unexpected (Zhang et al., 2007). The dominant photopeak of ^153^Sm is at 103 keV with an abundance of 29.8%. Gadolinium-153 has a photopeak at 97.4 keV with an abundance of 29.0% and another at 103 keV with an abundance of 21.1% (International Commission on Radiological Protection, 2008). This spectral overlap has been recognized as an analytical problem in the production of ^153^Gd when there is ^152^Sm present in the ^152^Gd target (Holden, 1986). In the case of a small amount of ^153^Gd in a sample of ^153^Sm, the photopeak of ^153^Sm overwhelms those of ^153^Gd, and thus ^153^Gd at low concentrations cannot be measured with gamma ray spectroscopy until the ^153^Sm has decayed to a negligible level compared to that of ^153^Gd. In a spark-source mass spectroscopic analysis of a sample of 99.47% enriched ^152^Sm that was commissioned by the authors, gadolinium was found with an upper limit of 200 ppm. This is the most likely source of the ^153^Gd impurity in ^153^Sm. The MURR higher-specific-activity sample was too old for any detectable ^153^Gd to remain and thus the amount was estimated based upon the irradiation conditions and the amount of ^153^Gd that had been measured in the lower-specific-activity ^153^Sm from MURR.

Tables 5 and 6 can be used to estimate the dosimetry of other preparations of ^153^Sm-DOTMP that might contain different concentrations of the impurities than the four sources of ^153^Sm that were analyzed here. Based upon the relative doses of the impurities to the dose from pure ^153^Sm, the preferred source for preparing ^153^Sm-DOTMP would be a lower-specific-activity ^153^Sm using a highly-enriched ^152^Sm target that had been activated for a relatively short time in a relatively low neutron flux.

At a dosage of 37 MBq/kg of ^153^Sm-DOTMP, i.e. 2.7 GBq for a 73 kg adult man and 2.2 GBq for a 60 kg adult woman, which is the standard dosage of ^153^Sm-EDTMP for bone pain palliation, the effective dose from the impurities in the MURR lower-specific-activity ^153^Sm to the adult man is 1.63 mSv or a background-equivalent radiation time (BERT) (Nickoloff et al., 2008) of about 160 days at sea level. For the adult woman, the effective dose from the impurities in the MURR lower-specific-activity ^153^Sm is 1.54 mSv or a BERT of about 150 days at sea level. What is more, since most of this dose is imparted over the course of 50 years, the dose rate is well below the natural background dose rate.

Past clinical uses of the radiopharmaceutical ^153^Sm-EDTMP, which must be prepared with higher-specific-activity ^153^Sm, have included high dosages of up to 50 GBq for the ablation of bone marrow (Bartlett et al., 2002) and fifteen or more administrations of low dosages such as 18.5 MBq/kg (0.5 mCi/kg) over the course of four years or longer for durable bone pain palliation and tumor treatment (Sinzinger et al., 2009). If the newer radiopharmaceutical ^153^Sm-DOTMP were to be used in similar fashions, the reduction in the doses to the target organs and the effective doses from the radionuclidic impurities in lower-specific-activity ^153^Sm compared to higher-specific-activity ^153^Sm would be even more favorable when the total administered activity of ^153^Sm is large.

## 5. Conclusion

The effective dose from the radionuclidic impurities in the lower-specific-activity ^153^Sm that is used to make ^153^Sm-DOTMP is a small fraction of the overall effective dose.

## Figures and Tables

**Table 1:** The half-lives and emitted energy (International Commission on Radiological Protection, 2008) (MeV/Bq-s) of ^153^Sm and its more prominent potential impurities. The photon energy combines gamma rays and X-rays. The charged particle radiation combines beta particles, internal conversion electrons and Auger electrons.

	^153^Sm	^145^Sm	^151^Sm	^153^Gd	^152^Eu	^154^Eu	^155^Eu	^156^Eu

**Half-life**	46.3 h	340 d	88.1 y	242 d	13.5 y	8.593 y	4.76 y	15.2 d
**Photon**	0.0643	0.0642	0.0000157	0.106	1.18	1.25	0.0612	1.23
**Charged particle**	0.270	0.0479	0.0200	0.0438	0.129	0.273	0.0647	0.458

**Table 2:** Relative activities of the radionuclidic impurities from the four production sources of Sm-153. Naseri et al. (2021) do not report the flux of their neutron source or the length of time over which their targets were activated, so these were inferred from (Naseri et al., 2011).. (SA = specific activity, NR = not reported, ND = not detected, and underlined = estimated.)

	Neutron Flux	Activ. Time	^153^Sm	^145^Sm	^151^Sm	^153^Gd	^152^Eu	^154^Eu	^155^Eu	^156^Eu

**Naseri Natural ^152^Sm**	5 × 10^13^ n/cm2-s	2 d	1.0	2.71 × 10^−5^	5.81 × 10^−5^	NR	1.46 × 10^−10^	6.43 × 10^−6^	1.81 × 10^−4^	8.50 × 10^−4^
**Naseri Enriched ^152^Sm**	5 × 10^13^ n/cm2-s	2 d	1.0	2.25 × 10^−7^	1.94 × 10^−7^	NR	4.97 × 10^−11^	3.14 × 10^−6^	6.94 × 10^−7^	3.41 × 10^−6^
**MURR Higher SA**	2.2 × 10^14^ n/cm^2^-s	6.5 d	1.0	ND	ND	3.76 × 10^−6^	7.69 × 10^−6^	3.85 × 10^−5^	5.32 × 10^−6^	3.48 × 10^−5^
**MURR Lower SA**	8 × 10^13^ n/cm^2^-s	48 h	1.0	ND	ND	6.38 × 10^−7^	7.21 × 10^−8^	2.35 × 10^−6^	3.50 × 10^−7^	1.45 × 10^−6^

**Table 3:** Time-integrated activity coefficients (in hours) for the ICRP 89 Adult Male model.

Adult Male Source Organ	^153^Sm	^145^Sm	^151^Sm	^153^Gd	^152^Eu	^154^Eu	^155^Eu	^156^Eu

Cortical Bone	13.45	2379	73230	1693	31860	21570	12160	106.3
Trabecular Bone	21.98	3882	119500	2763	51970	35200	19840	173.5
Blood	0.02681	0.02707	0.02707	0.02707	0.02707	0.02707	0.02707	0.02704
Heart	0.000441	0.000487	0.000487	0.000487	0.000487	0.000487	0.000487	0.000480
Lung	0.01297	0.02372	0.02385	0.02367	0.02384	0.02384	0.02382	0.02138
Muscle	0.08030	0.08198	0.08199	0.08198	0.08199	0.08199	0.08199	0.08178
Liver	0.05025	10.20	314.3	7.257	136.7	92.56	52.16	0.4472
Spleen	0.001612	0.002872	0.002885	0.002867	0.002884	0.002833	0.002882	0.002624
Kidney	0.1360	0.6334	0.6468	0.6281	0.6461	0.6457	0.6443	0.4356
Small Intestine	0.04942	0.05354	0.05357	0.05354	0.05357	0.05357	0.05357	0.05299
Right Colon	0.01802	0.01967	0.01968	0.01966	0.01968	0.01968	0.01968	0.01944
Left Colon	0.03604	0.03933	0.03935	0.03933	0.03935	0.03935	0.03935	0.03887
Rectum	0.01802	0.01967	0.01968	0.01966	0.01968	0.01968	0.01968	0.01944
Stomach	0.02450	0.02705	0.02707	0.02705	0.02707	0.02707	0.02707	0.02671

**Table 4:** Time-integrated activity coefficients (in hours) for the ICRP 89 Adult Female model.

Adult Female Source Organ	^153^Sm	^145^Sm	^151^Sm	^153^Gd	^152^Eu	^154^Eu	^155^Eu	^156^Eu

Cortical Bone	11.92	2105	64800	1498	28190	19090	10760	94.07
Trabecular Bone	19.45	3435	105700	2445	45990	31140	17550	153.5
Blood	0.02400	0.02423	0.02423	0.02423	0.02423	0.02423	0.02423	0.02421
Heart	0.00406	0.000449	0.000449	0.000448	0.000449	0.000449	0.000449	0.000442
Lung	0.01249	0.02285	0.02297	0.02280	0.02297	0.02296	0.02295	0.02060
Muscle	0.05896	0.06019	0.06020	0.06019	0.06020	0.06020	0.06020	0.06004
Liver	0.04755	9.652	297.4	6.867	129.3	87.59	49.36	0.4232
Spleen	0.00170	0.003028	0.003042	0.003023	0.003041	0.003040	0.003039	0.002767
Kidney	0.1442	0.6836	0.6981	0.6779	0.6973	0.6969	0.6954	0.4702
Small Intestine	0.04810	0.05211	0.05214	0.05211	0.05214	0.05214	0.05214	0.05158
Right Colon	0.02338	0.02552	0.02554	0.02552	0.02554	0.02554	0.02554	0.02523
Left Colon	0.04677	0.05105	0.05107	0.05104	0.05107	0.05107	0.05107	0.05047
Rectum	0.02338	0.02552	0.02554	0.02552	0.02554	0.02554	0.02554	0.02523
Stomach	0.04770	0.05265	0.05270	0.05265	0.05270	0.05270	0.05270	0.05199

**Table 5:** Target organ equivalent doses (in mSv/MBq) for the ICRP 89 Adult Male model for each of the radionuclides.

Adult Male Target Organ	^153^Sm	^145^Sm	^151^Sm	^153^Gd	^152^Eu	^154^Eu	^155^Eu	^156^Eu

Adrenals	0.00895	1.21	0.00228	1.46	293	211	6.14	1.03
Brain	0.00908	1.80	0.00387	1.86	334	240	7.24	1.11
Esophagus	0.00519	0.749	0.000616	0.987	210	152	4.33	0.711
Eyes	0.00908	1.80	0.00387	1.86	334	240	7.24	1.11
Gallbladder Wall	0.00300	0.306	0.000384	0.500	119	86.0	2.37	0.408
Left Colon	0.00432	0.796	0.00448	0.968	190	137	4.07	0.719
Small Intestine	0.00155	0.484	0.00138	0.690	146	106	3.11	0.518
Stomach Wall	0.00106	0.305	0.000898	0.447	108	78.0	2.04	0.387
Right Colon	0.00131	0.429	0.00133	0.603	132	94.9	2.71	0.473
Rectum	0.00256	0.789	0.00297	0.963	189	136	4.06	0.692
Heart Wall	0.00376	0.489	0.000451	0.694	150	109	3.13	0.512
Kidneys	0.0726	0.633	0.0246	0.846	172	125	3.52	1.01
Liver	0.00770	0.617	2.01	0.758	143	107	3.91	0.528
Lungs	0.00636	0.798	0.00127	0.948	183	132	3.97	0.616
Pancreas	0.00452	0.506	0.000308	0.760	171	124	3.50	0.579
Prostate	0.00471	0.198	0.0000547	0.392	102	74.1	1.94	0.372
Salivary Glands	0.00506	0.741	0.000607	0.978	209	151	4.29	0.705
Red Marrow	0.713	3.64	5.9	4.70	1100	1210	26.4	11.0
Osteogenic Cells	4.03	28.1	150	32.9	2260	2680	250	19.0
Spleen	0.00514	0.435	0.00262	0.606	135	98.1	2.70	0.480
Testes	0.00260	0.191	0.0000495	0.386	116	84.9	1.94	0.400
Thymus	0.00329	0.484	0.000710	0.615	135	98.0	2.66	0.464
Thyroid	0.00508	0.741	0.000607	0.979	209	151	4.30	0.706
Urinary Bladder	0.278	0.246	0.0201	0.453	102	74.4	2.01	0.922
Total Body	0.0880	3.82	30.5	3.63	349	309	23.8	1.88
Effective Dose	0.144	1.10	2.29	1.39	259	247	7.85	1.90

**Table 6:** Target organ equivalent doses (in mSv/MBq) for the ICRP 89 Adult Female model for each of the radionuclides.

Adult Female Target Organ	^153^Sm	^145^Sm	^151^Sm	^153^Gd	^152^Eu	^154^Eu	^155^Eu	^156^Eu

Adrenals	0.00985	1.49	0.00262	1.72	334	241	7.08	1.14
Brain	0.00965	1.94	0.00447	1.98	357	257	7.67	1.20
Breasts	0.00252	0.414	0.000816	0.470	110	79.9	1.93	0.385
Esophagus	0.00534	0.821	0.000849	1.03	227	165	4.40	0.778
Eyes	0.00965	1.94	0.00447	1.98	357	257	7.67	1.20
Gallbladder Wall	0.00368	0.354	0.000158	0.557	128	92.9	2.60	0.448
Left Colon	0.0523	0.927	000536	1.11	210	151	4.65	0.799
Small Intestine	0.0185	0.564	0.00170	0.783	161	117	3.49	0.578
Stomach Wall	0.0203	0.421	0.00174	0.589	126	91.1	2.63	0.472
Right Colon	0.0156	0.513	0.00164	0.684	139	101	3.00	0.506
Rectum	0.0329	0.926	0.00352	1.11	210	151	4.64	0.788
Heart Wall	0.00421	0.579	0.000689	0.780	160	115	3.45	0.540
Kidneys	0.0881	0.845	0.0300	1.08	201	146	4.41	1.09
Liver	0.00930	0.760	2.45	0.905	166	125	4.63	0.618
Lungs	0.00737	0.930	0.00144	1.08	200	144	4.50	0.674
Ovaries	0.00655	0.730	0.000606	0.950	180	129	4.13	0.618
Pancreas	0.00512	0.605	0.000538	0.880	180	130	4.00	0.615
Salivary Glands	0.00519	0.809	0.000830	1.01	226	164	4.35	0.770
Red Marrow	0.819	3.92	6.79	5.13	1240	1360	29.3	12.5
Osteogenic Cells	3.58	28.3	133	31.6	2130	2450	229	17.2
Spleen	0.00666	0.571	0.000784	0.752	154	111	3.28	0.552
Thymus	0.00365	0.554	0.000850	0.688	150	108	2.94	0.505
Thyroid	0.00521	0.809	0.000830	1.01	226	164	4.36	0.771
Urinary Bladder	0.360	0.324	0.0265	0.562	116	82.2	2.47	1.09
Uterus	0.00779	0.382	0.000137	0.620	135	97.5	2.90	0.495
Total Body	0.0955	4.25	32.8	3.98	379	334	25.8	2.03
Effective Dose	0.159	1.28	2.25	1.58	300	283	8.59	2.17

**Table 7:** The equivalent dose to each target organ from the ^153^Sm and impurities (mSv/MBq) and the equivalent dose from the impurities alone in each of the four sources of ^153^Sm for the ICRP 89 Adult Male model.

Adult Male Target Organ	Naseri Natural ^152^Sm Target		Naseri Enriched ^152^Sm Target		MURR Higher SA ^153^Sm		MURR Lower SA ^153^Sm	
				
	Total	Impurities	Total	Impurities	Total	Impurities	Total	Impurities

Adrenals	0.01233	0.00338	0.00962	0.00067	0.01940	0.01045	0.00947	0.00052
Brain	0.01293	0.00385	0.00984	0.00076	0.02097	0.01189	0.00967	0.00059
Esophagus	0.00758	0.00239	0.00567	0.00048	0.01271	0.00752	0.00556	0.00037
Eyes	0.01293	0.00385	0.00984	0.00076	0.02097	0.01189	0.00967	0.00059
Gallbladder Wall	0.00434	0.00134	0.00327	0.00027	0.00725	0.00425	0.00321	0.00021
Left Colon	0.04545	0.00225	0.04364	0.00044	0.04999	0.00679	0.04354	0.00034
Small Intestine	0.01720	0.00170	0.01584	0.00034	0.02074	0.00524	0.01576	0.00026
Stomach Wall	0.01183	0.00121	0.01085	0.00025	0.01446	0.00386	0.01079	0.00019
Right Colon	0.01461	0.00151	0.01340	0.00030	0.01780	0.00470	0.01333	0.00023
Rectum	0.02782	0.00222	0.02603	0.00043	0.03234	0.00674	0.02594	0.00034
Heart Wall	0.00548	0.00172	0.00411	0.00035	0.00915	0.00539	0.00403	0.00027
Kidneys	0.07492	0.00232	0.07300	0.00040	0.07879	0.00619	0.07291	0.00031
Liver	0.00968	0.00198	0.00804	0.00034	0.01296	0.00526	0.00796	0.00026
Lungs	0.00847	0.00211	0.00678	0.00042	0.01290	0.00654	0.00669	0.00033
Pancreas	0.00646	0.00194	0.00491	0.00039	0.01065	0.00613	0.00483	0.00031
Prostate	0.00586	0.00115	0.00495	0.00024	0.00837	0.00366	0.00489	0.00018
Salivary Glands	0.00743	0.00237	0.00554	0.00048	0.01253	0.00747	0.00543	0.00037
Red Marrow	0.7354	0.02235	0.7169	0.00386	0.7686	0.05558	0.7160	0.00295
Osteogenic Cells	4.118	0.08811	4.039	0.00658	4.153	0.1227	4.037	0.00658
Spleen	0.00668	0.00154	0.00545	0.00869	0.00999	0.00485	0.00538	0.00024
Testes	0.00384	0.00124	0.00287	0.00027	0.00679	0.00419	0.00281	0.00021
Thymus	0.00481	0.00152	0.00353	0.00031	0.00814	0.00485	0.00353	0.00024
Thyroid	0.00745	0.00237	0.00556	0.00048	0.01255	0.00747	0.00545	0.00037
Urinary Bladder	0.2796	0.00163	0.2782	0.00100	0.2817	0.00369	0.2782	0.00018
Total Body	0.09777	0.00479	0.08900	0.00079	0.1028	0.01479	0.08876	0.00076
Effective Dose	0.1488	0.00479	0.1448	0.00079	0.1556	0.01161	0.1446	0.0060

**Table 8:** The equivalent dose to each target organ from the ^153^Sm and impurities (mSv/MBq) and the equivalent dose from the impurities alone in each of the four sources of ^153^Sm for the ICRP 89 Adult Female model.

Adult Female Target Organ	Naseri Natural ^152^Sm Target		Naseri Enriched ^152^Sm Target		MURR Higher SA ^153^Sm		MURR Lower SA ^153^Sm	
				
	Total	Impurities	Total	Impurities	Total	Impurities	Total	Impurities

Adrenals	0.01369	0.00384	0.01062	0.00077	0.02178	0.01193	0.01044	0.00059
Brain	0.01376	0.00411	0.01047	0.00082	0.02238	0.01273	0.01028	0.00063
Breasts	0.00372	0.00120	0.00277	0.00025	0.00647	0.00395	0.00272	0.00020
Esophagus	0.00788	0.00254	0.00586	0.00052	0.01349	0.00815	0.00575	0.00041
Eyes	0.01376	0.00411	0.01047	0.00082	0.02238	0.01273	0.01028	0.00063
Gallbladder Wall	0.00514	0.00146	0.00398	0.00030	0.00827	0.00459	0.00391	0.00023
Left Colon	0.05482	0.0252	0.05278	0.00048	0.05978	0.00748	0.05267	0.00037
Small Intestine	0.02039	0.00189	0.01887	0.00037	0.02428	0.00578	0.01879	0.00029
Stomach Wall	0.02177	0.00147	0.02059	0.00029	0.02481	0.00451	0.02052	0.00022
Right Colon	0.01724	0.00164	0.01592	0.00032	0.02059	0.00499	0.01585	0.00025
Rectum	0.03541	0.00251	0.03338	0.00048	0.04038	0.00748	0.03327	0.00037
Heart Wall	0.00605	0.00184	0.00458	0.00037	0.00991	0.00570	0.00449	0.00028
Kidneys	0.09086	0.00276	0.08857	0.00047	0.09533	0.00723	0.08846	0.00036
Liver	0.01163	0.00233	0.00970	0.00040	0.01544	0.00614	0.00961	0.00031
Lungs	0.00971	0.00234	0.00783	0.00046	0.01450	0.00713	0.00773	0.00036
Ovaries	0.00867	0.00212	0.00696	0.00041	0.01295	0.00640	0.00687	0.00032
Pancreas	0.00722	0.00210	0.00553	0.00041	0.01155	0.00643	0.00544	0.00032
Salivary Glands	0.00771	0.00252	0.00571	0.00052	0.01330	0.00811	0.00559	0.00040
Red Marrow	0.8442	0.02517	0.8233	0.00433	0.8815	0.06250	0.8223	0.00602
Osteogenic Cells	3.660	0.08032	3.588	0.00794	3.693	0.1126	3.586	0.00331
Spleen	0.00845	0.00179	0.00701	0.00035	0.01216	0.00550	0.00693	0.00027
Thymus	0.00532	0.00167	0.00399	0.00034	0.00900	0.00535	0.00392	0.00027
Thyroid	0.00773	0.00252	0.00573	0.00052	0.01332	0.00811	0.00561	0.00040
Urinary Bladder	0.3619	0.00191	0.3603	0.00026	0.3641	0.00411	0.3602	0.00020
Uterus	0.00937	0.00158	0.00810	0.00031	0.01262	0.00483	0.00803	0.00024
Total Body	0.1061	0.01056	0.09658	0.00108	0.1115	0.01600	0.09633	0.00083
Effective Dose	0.1644	0.00538	0.1599	0.00090	0.1723	0.01333	0.1597	0.00069

**Table 9:** The times in hours after calibration to reach the USP limits of 9.3 × 10−5 for ^154^Eu in ^153^Sm and of 0.1907% for other impurities in ^153^Sm for the four sources of ^153^Sm.

	Naseri Natural ^152^Sm Target	Naseri Enriched ^152^Sm Target	MURR Higher SA ^153^Sm	MURR Lower SA ^153^Sm

^154^Eu-to-^153^Sm limit	85.74 h	108.8 h	28.3 h	118.1 h
Other impurities-to-^153^Sm limit	17.19 h	194.0 h	118.3 h	212.8 h

## Appendix. Time-Activity Curve Fitting Parameters

**Table 10:** Fitting parameters for the biological data decayed by the different radionuclides. The fits were either to a single exponential, a bi-exponential, or a trapezoidal approximation to the data followed by a single exponential with the physical half-life of the radionuclide when the last datum was not nil.

Source Organ		^153^Sm	^145^Sm	^151^Sm	^153^Gd	^152^Eu	^154^Eu	^155^Eu	^156^Eu

Bone	Y_0_	0.4075	0.4084	0.4084	0.4084	0.4084	0.4084	0.4084	0.4082
	T_eff_	46.3 h	340 d	88.1 y	242 d	13.5 y	8.593 y	4.76 y	15.2 d
Blood	Y_0_	0.04011	0.04022	0.04022	0.04022	0.04022	0.04022	0.04022	0.4021
	T_eff_	0.3483 h	0.3507 h	1.976 h	0.3507 h	0.3507 h	0.3507 h	0.3507 h	0.3504 h
Heart	Y_0_	6.777 × 10 ^5^	6.676 × 10 ^5^	6.676 × 10 ^5^	6.677 × 10 ^5^	6.676 × 10 ^5^	6.676 × 10 ^5^	6.676 × 10 ^5^	6.695 × 10 ^5^
	T_eff_	3.740 h	4.191 h	4.194 h	4.190 h	4.194 h	4.194 h	4.194 h	4.123 h
Lung	Y_0_	3.541 × 10 ^4^	3.464 × 10 ^4^	3.464 × 10 ^4^	3.464 × 10 ^4^	3.464 × 10 ^4^	3.464 × 10 ^4^	3.464 × 10 ^4^	3.470 × 10 ^4^
	T_eff0_	1.411 h	1.569 h	1.570 h	1.569 h	1.570 h	1.570 h	1.570 h	1.556 h
	Y_1_	1.232 × 10 ^4^	1.152 × 10 ^4^	1.152 × 10 ^4^	1.152 × 10 ^4^	1.152 × 10 ^4^	1.152 × 10 ^4^	1.152 × 10 ^4^	1.156 × 10 ^4^
	T_eff1_	21.31 h	44.91 h	45.17 h	44.80 h	45.16 h	45.15 h	45.12 h	39.88
Muscle	Y_0_	0.07589	0.07634	0.07634	0.07634	0.07634	0.07634	0.07634	0.07629
	T_eff_	0.8176 h	0.8298 h	0.8299 h	0.8298 h	0.8299 h	0.8299 h	0.8299 h	0.8283 h
Spleen	Y_0_	9.017 × 10 ^5^	8.786 × 10 ^5^	8.785 × 10 ^5^	8.787 × 10 ^5^	8.785 × 10 ^5^	8.785 × 10 ^5^	8.785 × 10 ^5^	8.817 × 10 ^5^
	T_eff_	15.94 h	29.14 h	29.28 h	29.09 h	29.27 h	29.26 h	29.25 h	26.53 h
Kidney	Y_0_	0.004688	0.004718	0.004718	0.004718	0.004718	0.004718	0.004718	0.004714
	T_eff_	38.21 h	180.0 h	183.8 h	178.5 h	183.6 h	183.5 h	183.1 h	123.9 h
Small Intestine	A	0.3059	0.3314	0.3316	0.3314	0.3316	0.3316	0.3316	0.3280
Large Intestine	A	0.5637	0.6153	0.6156	0.6152	0.6156	0.6156	0.6156	0.6083
Stomach	A	0.09404	0.1038	0.1039	0.1038	0.1039	0.1039	0.1039	0.1025
Liver	A	0.04145	0.06053	0.06067	0.06047	0.06086	0.06066	0.06064	0.05762
	Y_48_	0.000786	0.004718	0.004718	0.004718	0.004718	0.004718	0.004718	0.004714

Y_0_ and T_eff_ are the parameters of a single exponential fit, Y_0_, T_eff0_, Y_1_ and T_eff1_ are the parameters of a bi-exponential fit, and A is the area under the trapezoidal fit to the data out to 48 h followed by an exponential with the initial amplitude Y_48_, which is the last decayed measured datum, and the physical half-life of the radionuclide. The intestines and the stomach had no appreciable activity in the 48-h datum, hence just the area under the trapezoidal fit was used.
